# Binding Analysis of Human Immunoglobulin G as a Zinc-Binding Protein

**DOI:** 10.3390/antib5020013

**Published:** 2016-05-19

**Authors:** Yu Yamanaka, Sho Matsugano, Yasunaga Yoshikawa, Koichi Orino

**Affiliations:** Laboratory of Veterinary Biochemistry, School of Veterinary Medicine, Kitasato University, Aomori 034-8628, Japan; t-g-i-f@ezweb.ne.jp (Y.Y.); matstgan@outlook.jp (S.M.); yyoshikawa@vmas.kitasato-u.ac.jp (Y.Y.)

**Keywords:** immunoglobulin G, mammals, EDTA, fibrinogen, zinc ion

## Abstract

Human immunoglobulin G (IgG) binding with zinc ions was examined using zinc ions immobilized on chelating Sepharose beads (Zn-beads). Human IgG bound to Zn-beads but not to Sepharose beads (control beads). Mouse, rat, bovine and equine IgGs also bound to Zn-beads, similar to human IgG. The human IgG F(c) fragment showed zinc ion–binding activity whereas the Fab fragment did not. Ethylenediaminetetraacetic acid (EDTA)-treated Zn-beads no longer bound human IgG; however, washing the beads, followed by the addition of zinc ions, restored the binding activity towards human IgG. Zn-beads saturated with human fibrinogen could bind human IgG, and Zn-beads saturated with human IgG could bind fibrinogen. These results suggest that animal IgGs, including human, specifically bind zinc ions, probably through a zinc-binding site in the F(c) fragment and not in the Fab fragment. In addition, IgG and fibrinogen interact with each other and/or bind zinc ions through different mechanisms.

## 1. Introduction

It was recognized in 1869 that zinc is necessary for the growth of *Aspergillus niger* [1,2,3] and later its importance for the growth of plants and animals was documented [1], but it was not until 1961 that it was accepted that zinc deficiency could occur in humans [1,2,3]. Nutritional deficiency of zinc in humans occurs worldwide, particularly in areas where people eat cereal proteins containing a high concentration of organic phosphate compounds such as phytate, which hinder the absorption of zinc [1]. Zinc deficiency manifests as growth retardation, testicular and ovarian dysfunction, neurosensory disorders, immune dysfunction and cognitive impairment [1,2]. Zinc administration improves these syndromes and zinc acts as an antioxidant and anti-inflammatory agent [1,2,3,4].

Immune functions are very sensitive to zinc restriction [2]. Zinc is essential for T cell differentiation, suggesting that it affects the up-regulation of mRNAs of factors such as IFN-γ, IL-12 receptor β_2_ and T-bet [5]. High concentrations of zinc inhibit the production of pro-inflammatory cytokines in monocytes/macrophages, resulting in the down-regulation of TNF-α, IL-1 and IL-6 [6]. Zinc relieves oxidative stress by acting as an inhibitor of NADPH oxidase and the co-factor of super oxide dismutase, and by inducing metallothionein production [1,2]. Furthermore, zinc supplementation augments the antitumor effect of tumor chemotherapy by enhancing p53 function [7].

Homeostasis of the intracellular zinc level is strictly regulated by the zinc transporter [8]. There are many zinc-binding proteins in human blood such as albumin, α_2_-macroglobuin, haptoglobulin, ceruloplasmin, immunoglobulins (IgG, IgM and IgA), complement C_4_, prealbumin, C-reactive protein, and fibrinogen [9,10,11,12,13]. Zinc-binding proteins may act as zinc storage compounds for maintaining immunoregulatory and oxidative balance [10]. IgG is believed to preferentially change conformation to allow for zinc transport through its zinc-binding ability and to distribute zinc ions in the cell [11]. 

A number of zinc ion binding proteins have been identified, and the cellular uptake of zinc ions, the effect of zinc ion uptake on cellular function, and the essential need of immune cells and enterocytes for zinc have been revealed. However, the binding mechanism of zinc ions by circulating zinc ion binding proteins remains unclear. This study presents a binding analysis of zinc ions with human IgG and speculates on the zinc-binding form of the protein in circulation.

## 2. Results and Discussion

### 2.1. Binding of Mammalian IgGs to Zn-Beads

**Figure 1:**
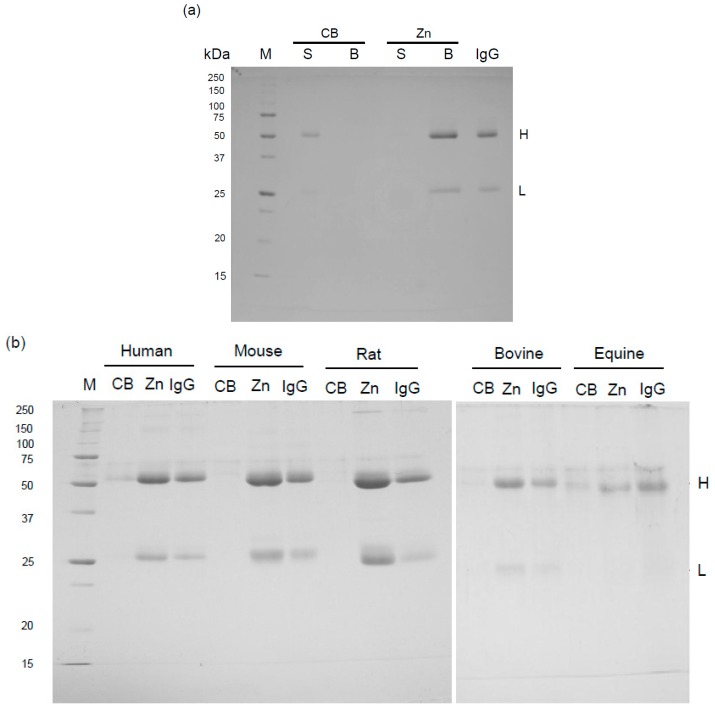
Binding of human and animal IgGs to Zn-beads. (**a**) Aliquots (1 mL) of IgG (25 µg) and Zn-beads (Zn) or CB in phosphate-buffered saline (PBS) were prepared (net volume of beads per sample: 20 µL each) and incubated at 4 °C overnight. The mixture was centrifuged at 14,000× *g* for 7 min and the supernatant (S) and pelleted beads (B) were analyzed by sodium dodecyl sulfate-polyacrylamide gel electrophoresis (SDS-PAGE) as described in the “Experimental Section”. Human IgG (IgG) was also applied to the gel (2 µg/lane) and the separated H and L subunits are denoted H and L, respectively. M represents marker proteins; (**b**) Animal IgG samples were treated as described in (**a**), and only the resultant beads were analyzed by SDS-PAGE. The H and L subunits from the various animal IgGs (2 µg each per lane) were denoted H and L. M represents marker proteins.

**Figure 2:**
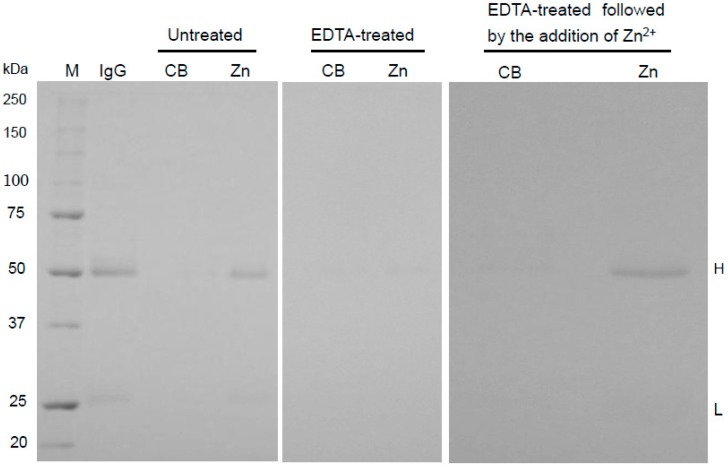
Effect of ethylenediaminetetraacetic acid (EDTA) on the binding of human IgG to Zn-beads. Zn-beads and CB were treated with or without 50 mM EDTA (pH 7.4). After washing, PBS (980 µL) containing human IgG (25 µg) was added to aliquots of the treated and untreated Zn-beads and CB (net volume of beads per sample: 20 µL). Different aliquots of EDTA-treated CB and Zn-beads were washed and incubated with 1 mL 50 mM ZnSO_4_, rotated overnight, and then the ZnSO_4_-treated beads were washed and suspended at 50% (*v*/*v*) in PBS. Aliquots (1 mL) of IgG (25 µg) and ZnSO_4_-treated beads (Zn-beads or CB) in PBS were prepared (net volume of beads per sample: 20 µL each), and incubated at 4 °C overnight. As described in the “Experimental Section”, the obtained bead sample was analyzed by SDS-PAGE; human IgG (IgG) was also applied to the gel (2 µg/lane), and the separated H and L subunits were denoted H and L, respectively. M represents marker proteins.

Human IgG was incubated with zinc ion immobilized on chelating Sepharose beads (Zn-beads) or Sepharose beads (control beads: CB), and then the suspension was centrifuged. Human IgG was detected by sodium dodecyl sulfate-polyacrylamide gel electrophoresis (SDS-PAGE) analysis in the supernatant of CB but not Zn-beads (Figure 1a): the CB supernatant showed two bands corresponding to the H (55 kDa) and L (23 kDa) subunits of human IgG comigrated. In the Zn-beads supernatant, the IgG H and L subunit bands were detected in the pelleted beads, indicating the binding of human IgG to zinc ions. On the other hand, natural antibodies such as anti-carbohydrate antibodies are found in normal human serum [14], and, as described below, when CB was used, some of the IgG proteins could be detected by the interaction with the carbohydrate chain in the CB rather than its precipitation by centrifugation due to insufficient washing. Mouse, rat, bovine and equine IgGs also showed zinc ion–binding activity (Figure 2b). Animal IgGs, including human, were slightly detected in the pelleted CBs, probably due to insufficient washing of the beads and non-specific binding and/or carbohydrate binding of IgG to CB. The intensity of the Coomassie staining of IgG is species-dependent (Figure 1b). For example, equine IgG H and L subunits were less stained as compared with the IgG from other mammals, but a part of the IgG molecule appears to recognize the carbohydrate chain immobilized on the beads. The presence of a band with a higher molecular weight than the H subunit band in IgG from each mammal seems to be an artifact. These results indicate that mammalian IgGs have similar zinc ion binding activities.

### 2.2. Binding Mechanism of Human IgG to Zn-Beads

The zinc-binding activity of human IgG to EDTA-treated Zn-beads was decreased compared to its binding to untreated beads (Figure 2). The removal of EDTA and the addition of zinc ions to the beads restored the IgG-binding activity to the Zn-beads. EDTA also removed IgG bound to the Zn-beads (data not shown). This study demonstrates that the binding of IgG to Zn-beads is zinc ion–dependent.

**Figure 3:**
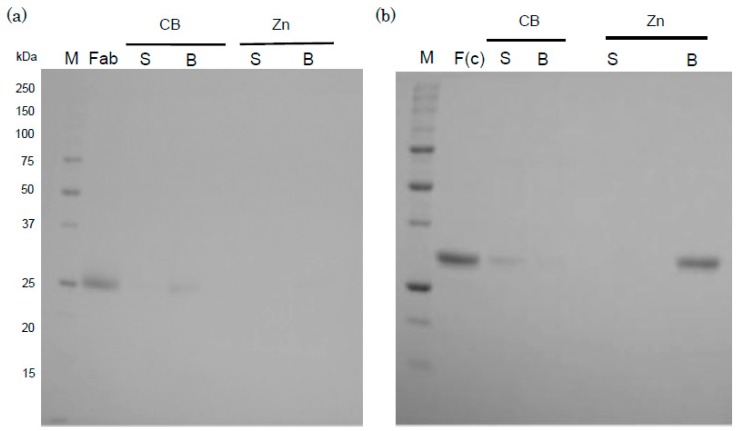
The binding of human IgG Fab (**a**) and F(c) fragments (**b**) to Zn-beads. Aliquots (1 mL) of each fragment (25 µg) and Zn-beads or CB in PBS were prepared (net volume of beads per sample: 20 µL each), and incubated at 4 °C overnight. The mixtures were centrifuged at 14,000× *g* for 7 min, and the supernatant and pelleted beads were subjected to SDS-PAGE as described in the “Experimental Section”. Supernatant (S) and pelleted beads (B) from respective CB and Zn-beads (Zn) incubated with Fab (**a**) and F(c) (**b**) were applied to SDS-PSGE. Human IgG Fab and F(c) fragment samples were also applied to the gel (2 µg each/lane). M represents marker proteins.

The binding of mammalian IgGs, including those of humans, to zinc ions is a common characteristic of IgGs, and therefore the binding of the Fab and F(c) fragments of human IgG to zinc ions was examined (Figure 3a,b). Human IgG Fab and F(c) fragments were detected by SDS-PAGE at very low levels in pelleted CB; in contrast, the F(c) fragments were strongly detected in a sample of pelleted Zn-beads but the Fab fragment was not, suggesting that the F(c) fragment is likely involved in the binding between IgG and zinc ions. Human anti-ferritin autoantibodies (IgG, IgM, IgA) bind with zinc ions [12], indicating that all classes of immunoglobulins bind zinc ions. Further studies are required to clarify the binding between zinc ions and the F(c) fragments of other mammalian IgGs, and the binding of zinc ions with other classes of human immunoglobulins.

### 2.3. Binding of Human IgG and Fibrinogen to Zinc Ions

**Figure 4:**
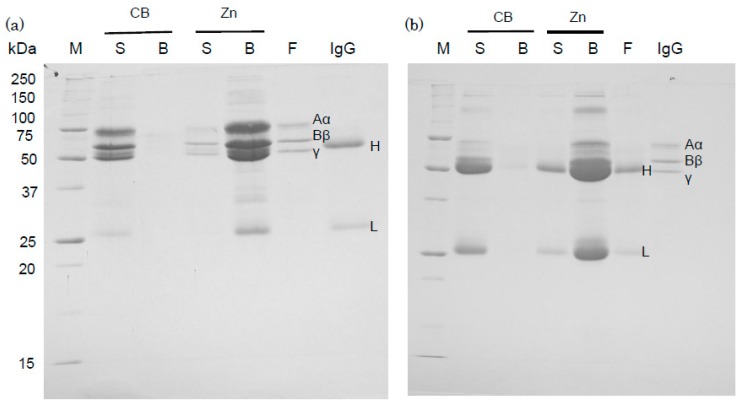
Binding of human IgG and fibrinogen to Zn-beads which were saturated with fibrinogen and IgG, respectively. After binding of human fibrinogen (600 µg) or IgG (500 µg) to Zn-beads or CB (net volume of beads per sample: 20 µL each), aliquots (20 µL) of human IgG (100 µg) or fibrinogen (100 µg) in PBS were added to protein-saturated Zn-beads and incubated with: fibrinogen in (**a**) and IgG in (**b**). As described in the “Experimental Section”, the supernatant (S) and bead samples (B) obtained after centrifugation were subjected to SDS-PAGE. Human fibrinogen (F) and IgG samples were also applied (2 µg/lane). Their separated subunit bands derive from fibrinogen (Aα, Bβ and γ) and IgG (H and L). M represents marker proteins.

Zn-beads were saturated with human fibrinogen; after centrifugation, unbound fibrinogen was detected in the supernatant (data not shown). In addition, human IgG was added to a suspension containing Zn-beads saturated with fibrinogen and containing unbound fibrinogen. Human fibrinogen was separated by SDS-PAGE into three bands corresponding to the Aα, Bβ and γ subunits, with molecular masses of 67 kDa, 56 kDa and 50 kDa, respectively, as shown in purified sample (F). The IgG H band appeared to comigrate with the γ band of fibrinogen present in the supernatant (S) of CB and present in the pelleted Zn-beads (B), as shown in Figure 4a. Fibrinogen bands were detected in the supernatant of Zn-beads whereas the IgG L band was not. This study demonstrates that the addition of human IgG to a suspension containing Zn-beads saturated with fibrinogen and containing unbound fibrinogen results in human IgG binding to the Zn-beads, even though they are saturated with fibrinogen. Zn-beads saturated with IgG were used to examine whether fibrinogen binds to IgG-bound beads. A strongly stained IgG H subunit band made it impossible to identify the Bβ and γ bands of human fibrinogen (Figure 4b) but the Aα band of fibrinogen was detected specifically in the supernatant (S) of CB and pelleted Zn-beads (B). IgG bands were also detected in the supernatant of Zn-beads but fibrinogen bands were not. The binding of human fibrinogen to zinc ions was previously studied using fibrinogen-immobilized Sepharose beads [13] and it was also shown that human fibrinogen binds to zinc ion–immobilized beads and is thus a zinc-binding protein. Interestingly, IgG and fibrinogen were detected bound to Zn-beads even after the addition to Zn-beads saturated with fibrinogen and IgG, respectively. In the present study, the addition of IgG and fibrinogen to beads was performed using a solution containing unbound proteins and the Zn-beads were pre-saturated with fibrinogen and IgG, respectively, because these bound proteins were removed from the beads by washing (data not shown). These results suggest that IgG molecules bind fibrinogen [15], and that these proteins bind zinc ions through two different mechanisms. Further study is needed to examine whether the binding activities of these proteins with zinc ions may change depending on the pH, solvent, chelator and spacer [16].

Zinc ions affect thrombin absorption with fibrin and shorten blood clotting time [17]. Fibrinogen interacts with IgG and enhances IgG-mediated phagocytosis [15]. This study also demonstrated the interaction between IgG and fibrinogen. However, it remains unclear whether IgG and fibrinogen compete in the circulation to bind to zinc ions. Fibrinogen is an antioxidant and is susceptible to oxidative stress [18,19,20]. Oxidative modifications of fibrinogen cause structural changes, suggesting that the zinc-binding activity of fibrinogen changes according to oxidative conditions in the circulation [20]. Fibrinogen binds iron and copper ions which cause oxidative stress [13,18,19,20]. Further study is required to clarify whether zinc ions compete with iron or copper ions for the zinc-binding site on the surface of fibrinogen [1,2].

Zinc plays multiple roles in animals, including humans [1,2,3]. More than 300 enzymes require zinc ions, and there are three distinct zinc-binding motifs in DNA-binding protein domains: zinc-fingers, zinc-twists, and zinc clusters [1,2,3]. Zinc functions as an antioxidant and anti-inflammatory agent through various mechanisms [1,2,3,4,18,19,20]. Many zinc-binding proteins are present in mammalian circulatory systems including albumin, α_2_-macroglobuin, haptoglobulin, ceruloplasmin, immunoglobulins (IgG, IgM and IgA), complement C_4_, prealbumin, C-reactive protein, and fibrinogen [9,10,11,12,13,14]. These proteins likely transport zinc ions to cells requiring zinc ions. Further study is required to elucidate the mechanism of binding between zinc ions and these proteins, and how zinc ions are taken up into cells through the interaction between these zinc-binding proteins. 

Zinc supplementation is effective in relieving oxidative stress and in decreasing the levels of pro-inflammatory cytokines such as TNF-α, IL-6 and IL-10 [1,2,3,4,6]. The clinical effects of zinc ions are very impressive and have a major impact on human health [1,2,3,21]. The present study suggests that IgG and fibrinogen interact with each other and/or bind zinc ions with different mechanisms. Zinc ions are taken up intracellularly by transporters but may also be indirectly taken up by receptors for zinc-binding proteins [22]. Further research is required to elucidate how important variables such as body condition, aging, species specificity and various zinc-binding proteins affect zinc availability.

## 3. Experimental Section

### 3.1. Chemicals

Human and rat IgGs were purchased from Invitrogen Corp. (Carisbad, CA, USA). Human IgG Fab and F(c) fragements and equine IgG were purchased from Rockland Immunochemicals Inc. (Limerick, PA, USA). Human fibrinogen and bovine IgG were purchased from Sigma (St. Louis, MO, USA). Mouse IgG was purchased from Innovative Research Inc. (Novi, MI, USA). Chelating Sepharose^TM^ Fast Flow and Sepharose 4B were purchased from GE Healthcare (Cleveland, OH, USA). Other analytical grade chemicals were purchased from Wako Pure Chemical Industries Ltd. (Osaka, Japan). Pure water (Elix water) was produced from tap water using an Elix Advantage Water Purification System (EMD Millipore, Billerica, MA, USA).

### 3.2. SDS-PAGE

SDS-PAGE was performed essentially according to the method of Laemmli [23] using slab gels consisting of a 4.5% polyacrylamide stacking gel and a 12% polyacrylamide running gel.

### 3.3. Binding of Mammalian IgGs to Zinc Ion–Immobilized Beads

Zinc ion was immobilized to Chelating Sepharose Fast Flow beads using 0.2 M ZnSO_4_ according to the manufacturer’s instructions and the beads were suspended to 50% (*v*/*v*) in phosphate-buffered saline (PBS, 150 mM NaCl, 20 mM sodium phosphate, pH 7.2). One mL PBS containing IgG, human IgG Fab or IgG F(c) fragments (25 µg each) and a suspension of 40 µL 50% (*v*/*v*) zinc ion–immobilized (Zn-beads) or Sepharose 4B (CB: control beads) beads was prepared (net volume of beads per sample: 20 µL) and the mixture was rotated at 4 °C overnight. The mixture was centrifuged at 14,000× *g* for 7 min at 4 °C, providing supernatant and pelleted beads for SDS-PAGE. One mL of washing solution (0.5 M NaCl, 20 mM sodium phosphate buffer, pH 7.2) was added to the pelleted beads and the suspension was centrifuged as described above. One mL of washing solution was then added, the beads were suspended, and centrifugation was repeated to further wash the beads. This washing step was repeated three times in total and the final pelleted beads were suspended in sample buffer for SDS-PAGE and used for SDS-PAGE (net volume of beads per lane: 8 µL).

The effect of EDTA on the binding between human IgG and Zn-beads was investigated by preincubating Zn-beads and CB in the presence and absence of 1 mL of 50 mM EDTA (pH 7.4), then IgG was added and the beads were incubated at 4 °C overnight. The treated and untreated beads were exhaustively washed as described above and the beads were suspended to provide a 50% (*v*/*v*) suspension in PBS. Human IgG was also incubated with treated and untreated Zn-beads and CB as previously described. In another experiment, the EDTA-treated Zn-beads or CB were exhaustively washed, then 1 mL PBS containing 50 mM ZnSO_4_ was added to the pelleted beads. The mixture was rotated at 4 °C overnight, then the beads were exhaustively washed, suspended to 50% (*v*/*v*) with PBS, and incubated with IgG (25 µg) as described above. In all experiments, beads were washed and centrifuged, the resulting supernatant and the pelleted beads were treated as described above, and these samples were subjected to SDS-PAGE.

### 3.4. Comparison of Binding Affinity of Human Fibrinogen and IgG to Zn-Beads

One mL PBS containing human fibrinogen (600 µg) or IgG (500 µg) and a suspension of 40 µL 50% (*v*/*v*) Zn-beads or CB beads was prepared (net volume of beads per sample: 20 µL) and the mixture was rotated at 4 °C overnight. The presence of human fibrinogen or IgG was detected in the supernatant, suggesting that the Zn-beads were saturated with fibrinogen or IgG (data not shown). A 20 microliter aliquot of human IgG (100 µg) and fibrinogen (100 µg) in PBS were added to a suspension of beads saturated with fibrinogen and IgG, respectively, and further incubated as described above. The mixture was centrifuged at 14,000× *g* for 7 min at 4 °C to provide supernatant and pelleted beads for SDS-PAGE. Pelleted beads were applied to gel as much as possible due to natural sedimentation. CB were treated and analyzed in an identical manner.

## 4. Conclusions

Animal IgGs, including humans, bind with zinc ions immobilized on Sepharose beads (Zn-beads). The human IgG F(c) fragment bound with Zn-beads whereas the Fab fragment did not, suggesting that IgG binds Zn ions through the F(c) fragment. EDTA-treated Zn-beads could not bind human IgG, but regeneration with zinc ions recovered the zinc-binding activity with human IgG. Human IgG retains zinc-binding activity even after addition to Zn-beads saturated with fibrinogen and fibrinogen binds to Zn-beads saturated with IgG. These results demonstrate that human IgG specifically binds zinc ions, suggesting a zinc-binding site in the Fc fragment, and IgG binds fibrinogen and/or zinc ions through a different mechanism.
